# Computational exploration of acefylline derivatives as MAO-B inhibitors for Parkinson’s disease: insights from molecular docking, DFT, ADMET, and molecular dynamics approaches

**DOI:** 10.3389/fchem.2024.1449165

**Published:** 2024-10-08

**Authors:** Ali Irfan, Ameer Fawad Zahoor, Yassir Boulaamane, Sadia Javed, Huma Hameed, Amal Maurady, Muhammed Tilahun Muhammed, Sajjad Ahmad, Aamal A. Al-Mutairi, Irum Shahzadi, Sami A. Al-Hussain, Magdi E. A. Zaki

**Affiliations:** ^1^ Department of Chemistry, Government College University Faisalabad, Faisalabad, Pakistan; ^2^ Laboratory of Innovative Technologies, National School of Applied Sciences of Tangier, Abdelmalek Essaadi University, Tetouan, Morocco; ^3^ Department of Biochemistry, Government College University Faisalabad, Faisalabad, Pakistan; ^4^ Faculty of Pharmaceutical Sciences, University of Central Punjab, Lahore, Pakistan; ^5^ Department of Pharmaceutical Chemistry, Faculty of Pharmacy, Suleyman Demirel University, Isparta, Türkiye; ^6^ Department of Health and Biological Sciences, Abasyn University Peshawar, Peshawar, Pakistan; ^7^ Gilbert and Rose-Marie Chagoury School of Medicine, Lebanese American University, Beirut, Lebanon; ^8^ Department of Natural Sciences, Lebanese American University, Beirut, Lebanon; ^9^ Department of Chemistry, College of Science, Imam Mohammad Ibn Saud Islamic University (IMSIU), Riyadh, Saudi Arabia

**Keywords:** acefylline derivatives, Parkinson’s disease, computer-aided drug design approach, MAO-B inhibitors, molecular docking, molecular dynamics simulations, ADMET, density functional theory studies

## Abstract

Monoamine oxidase B (MAO-B) plays a pivotal role in the deamination process of monoamines, encompassing crucial neurotransmitters like dopamine and norepinephrine. The heightened interest in MAO-B inhibitors emerged after the revelation that this enzyme could potentially catalyze the formation of neurotoxic compounds from endogenous and exogenous sources. Computational screening methodologies serve as valuable tools in the quest for novel inhibitors, enhancing the efficiency of this pursuit. In this study, 43 acefylline derivatives were docked against the MAO-B enzyme for their chemotherapeutic potential and binding affinities that yielded GOLD fitness scores ranging from 33.21 to 75.22. Among them, five acefylline derivatives, namely, **MAO-B14**, **MAO-B15**, **MAO-B16**, **MAO-B20**, and **MAO-B21**, displayed binding affinities comparable to the both standards istradefylline and safinamide. These derivatives exhibited hydrogen-bonding interactions with key amino acids Phe167 and Ile197/198, suggesting their strong potential as MAO-B inhibitors. Finally, molecular dynamics (MD) simulations were conducted to evaluate the stability of the examined acefylline derivatives over time. The simulations demonstrated that among the examined acefylline derivatives and standards, **MAO-B21** stands out as the most stable candidate. Density functional theory (DFT) studies were also performed to optimize the geometries of the ligands, and molecular docking was conducted to predict the orientations of the ligands within the binding cavity of the protein and evaluate their molecular interactions. These results were also validated by simulation-based binding free energies *via* the molecular mechanics energies combined with generalized Born and surface area solvation (MM-GBSA) method. However, it is necessary to conduct *in vitro* and *in vivo* experiments to confirm and validate these findings in future studies.

## 1 Introduction

**FIGURE 1:**
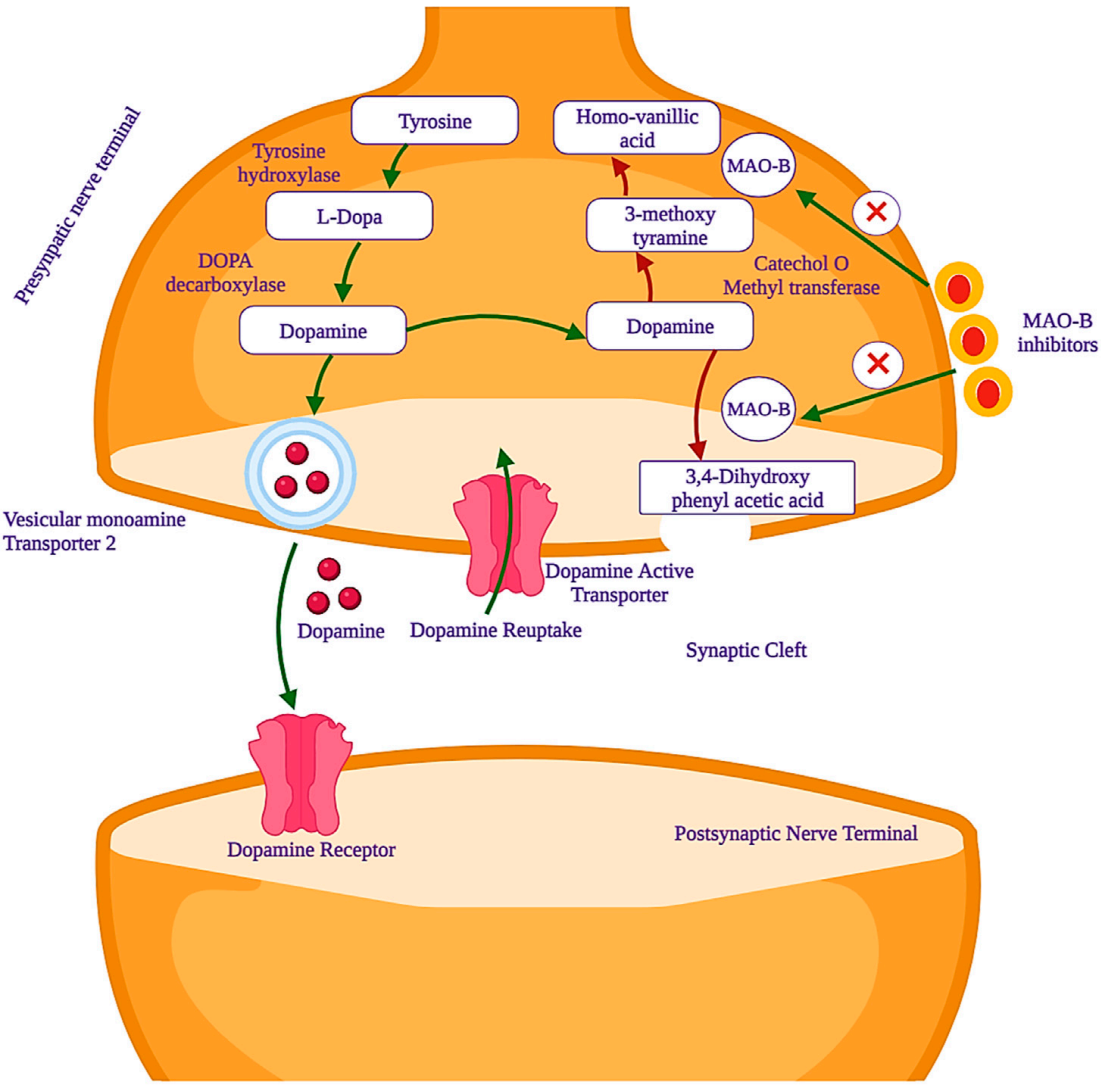
Mechanism of action of monoamine oxidase-B (MAO-B) inhibitors, which are used as potential drug candidates in the treatment of PD.

Parkinson’s disease (PD) is marked by the progressive degeneration of dopaminergic neurons, especially in the substantia nigra *pars compacta* of the midbrain (Armstrong and Okun, 2020a). Its global prevalence is estimated to impact 6 million individuals, with an incidence rate of 150 per 100,000 people, a figure projected to double or triple by 2030 (Pringsheim et al., 2014). It ranks as the second-most common neurodegenerative disorder, second only to Alzheimer’s disease (Yan et al., 2013). Current treatment options for PD include levodopa as the primary choice, along with dopamine agonists and catechol-O-methyl transferase (COMT) or monoamine oxidase (MAO) inhibitors (Armstrong and Okun, 2020b). MAO (EC 1.4.3.4) is a significant and crucial flavoenzyme that resides on the outer mitochondrial membrane of neurons and plays a pivotal and central role in the oxidative deamination of key monoamine neurotransmitters that exist in the central nervous system (CNS). The neurotransmitters are noradrenaline, adrenaline, and dopamine, which are essential and vital for various functions of the CNS (Riederer and Laux, 2011). From the blood–brain barrier (BBB), tyrosine is transferred and converted to levodopa by tyrosine hydroxylase (TH) and levodopa to dopamine by the aromatic amino acid decarboxylase (AADC) enzyme. The formed dopamine remains stored until it activates the striatum neurons linked to the dopamine receptors after releasing it in the synaptic cleft. The monoamine oxidase-B (MAO-B) enzyme is responsible for metabolizing the freely available dopamine to 3,4-dihydroxy phenylacetic acid (DOPAC), which is further converted to homovanillic acid (HMV) by COMT. The dopamine precursor, levodopa, is preferably used for the treatment of PD. Levodopa, after administration, can cross the BBB and will be converted to dopamine by the dopa decarboxylase (DDC) enzyme. This enzyme is present in the substantia nigra-linked presynaptic neurons. Overall, this treatment controls body movements and motor activity by reducing the muscle tones that are faced by PD patients. However, this important binding of dopamine-to-dopamine receptors quickly unbinds due to a dopamine transporter (DAT) and leads to the metabolism of dopamine (Cohen et al., 1997). At this stage, MAO inhibitors, especially MAO-B, play an important role in the prevention of dopamine metabolism. The activity of mono-amine oxidase-B inhibitors is shown in Figure 1.

The MAO enzyme has two isoforms, MAO-A and MAO-B, which share approximately 70% sequence homology but differ in substrate specificity, inhibitor selectivity, and tissue distribution (Baweja et al., 2023). MAOs are found in all human tissues, but MAO-A is primarily present in organs such as the gastrointestinal tract (GIT), placenta, and heart, while MAO-B is mainly found in platelets and glial cells in the brain. The early development of MAO inhibitors was halted due to the “cheese effect,” a problem with tyramine metabolism that led to cardiovascular crises (Armstrong and Okun, 2020b; Da Prada et al., 1988).

Nevertheless, a new generation of selective MAO-B-inhibiting agents has demonstrated efficacy in alleviating PD symptoms, and these inhibitors do not cause any kind of adverse effects (Armstrong and Okun, 2020b; Alborghetti and Nicoletti, 2019). MAO-A primarily metabolizes serotonin, while MAO-B preferentially deaminates 2-phenylethylamine and benzylamine. In most animal tissues, both isoforms are involved in the metabolism of dopamine, norepinephrine, and epinephrine (Carradori et al., 2018). With advancing age, MAO-B expression is upregulated in the brain, correlated with heightened dopamine metabolism, resulting in the generation of reactive oxygen species (ROS) like hydrogen peroxide (H_2_O_2_), culminating in oxidative damage and apoptotic signaling events (Fang et al., 2017). Experimental studies have demonstrated the impact of MAO-B inhibitors in reducing oxidative stress and neurodegeneration. For instance, selegiline, an irreversible MAO-B inhibitor, has been shown to alleviate motor symptoms in PD patients and reduce oxidative stress markers (Marconi and Zwingers, 2014; Wang et al., 2023). Similarly, rasagiline has demonstrated neuroprotective effects in animal models of PD, with significant improvements in motor function and reductions in oxidative damage (Dias et al., 2013). The latest approved MAO-B inhibitor, safinamide, operates through reversible inhibition, with an IC_50_ value of 450 nM and a selectivity index exceeding 700 (Wasan et al., 2021). Safinamide has been shown to exhibit neuroprotective effects in clinical studies (Teixeira et al., 2018). Recently, istradefylline, approved as an adenosine A_2A_ receptor antagonist, exhibits a dual mechanism of action by also acting as an MAO-B inhibitor, making it a promising candidate for treating PD. Although istradefylline demonstrated modest MAO-B inhibitory activity (IC_50_ = 28 μM), these findings emphasize the potential for exploring novel substitutions to enhance the xanthine core’s efficacy (Boulaamane et al., 2022). Clinical trials have demonstrated that istradefylline significantly reduces the “off” time in patients with PD. In several randomized, double-blind, placebo-controlled studies, istradefylline was shown to decrease the daily “off” time by approximately 0.75–0.82 h, depending on the dosage (20 mg or 40 mg) used (Cummins and Cates, 2022; Müller, 2023). These interpretations suggest that istradefylline can effectively augment the therapeutic effects of levodopa, especially in patients who are not optimally managed with dopaminergic medications alone.

**FIGURE 2:**
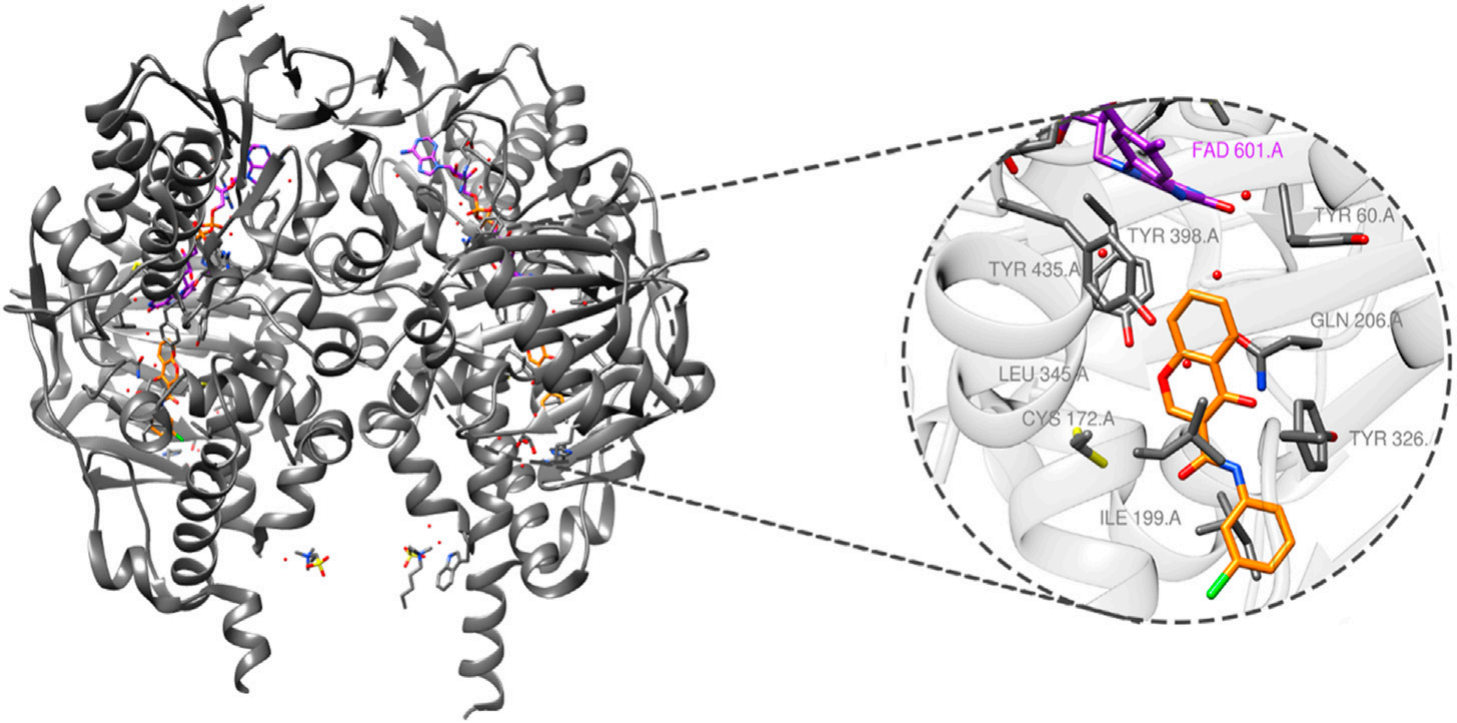
MAO-B structure (PDB ID: 6FW0) bound to chlorophenyl–chromone–carboxamide and its binding site.

MAO-B (PDB ID: 6FW0) is made up of two monomers, each of which has a globular domain attached to the membrane by a C-terminal helix, as demonstrated by structural analysis (Boulaamane et al., 2023a). The critical residues that define the active site, which are essential for substrate binding, are tyrosine-60 (Tyr-60), proline-102 (Pro-102), proline-104 (Pro-104), leucine-164 (Leu-164), phenylalanine-168 (Phe-168), leucine-171 (Leu-171), cysteine-172 (Cys-172), isoleucine-198 (Ile-198), isoleucine-199 (Ile-199), glutamine-206 (Gln-206), isoleucine-316 (Ile-316), tyrosine-326 (Tyr-326), phenylalanine- 343 (Phe-343), tyrosine-398 (Tyr-398), and tyrosine-435 (Tyr-435), as illustrated in Figure 2.

**FIGURE 3:**
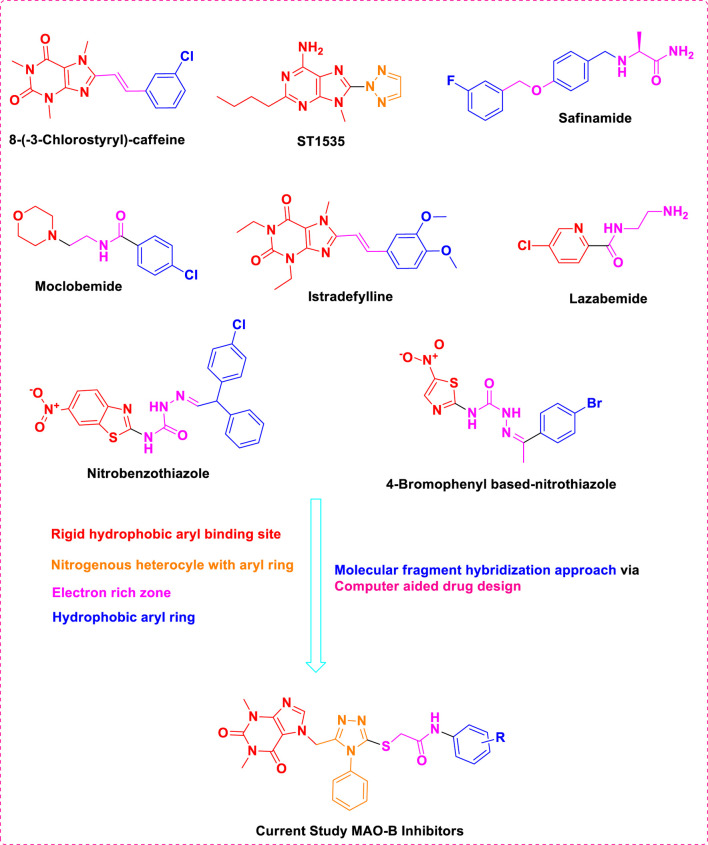
Structures of standard and lead MAO inhibitors and the rational design of the current study.

The previous literature on xanthine derivatives, as well as nitrogenous heterocyclic compounds, encouraged us to develop a rational design of acefylline derivatives to screen against the MAO-B enzyme to discover new therapeutic agents against neurodegenerative diseases. Our screened acefylline derivatives possessed all therapeutic fragments according to a fragment-based drug discovery (FBDD) approach, such as an electron-rich zone, hydrophobic aromatic ring (aryl-binding site as the entrance cavity), and rigid hydrophobic aryl-binding site as the substrate cavity (Osmaniye et al., 2021), which are crucial to display MAO-B inhibition activity, as present in lead and standard MAO inhibitors such as 8-(-3-chlorostyryl)-caffeine (CSC), substituted triazole moiety containing purin-6-amine (ST1535) (Rivara et al., 2013), safinamide (fluorobenzyl-based-*L*-alaninamide), moclobemide (chloro-moiety containing morpholin-4-yl)ethyl]benzamide), istradefylline (dimethoxyphenyl moiety containing dihydro-1*H*-purine-2,6-dione), lazabemide (aminoethyl moiety containing chloro-pyridine-2-carboxamide), chlorophenyl-based-nitrobenzothiazol-2-yl)semicarbazide, and bromophenyl-based-nitrothiazol-2-yl)semicarbazide) (Osmaniye et al., 2021; Silvestri et al., 2003; Tripathi et al., 2018), as depicted in Figure 3.

**FIGURE 4:**
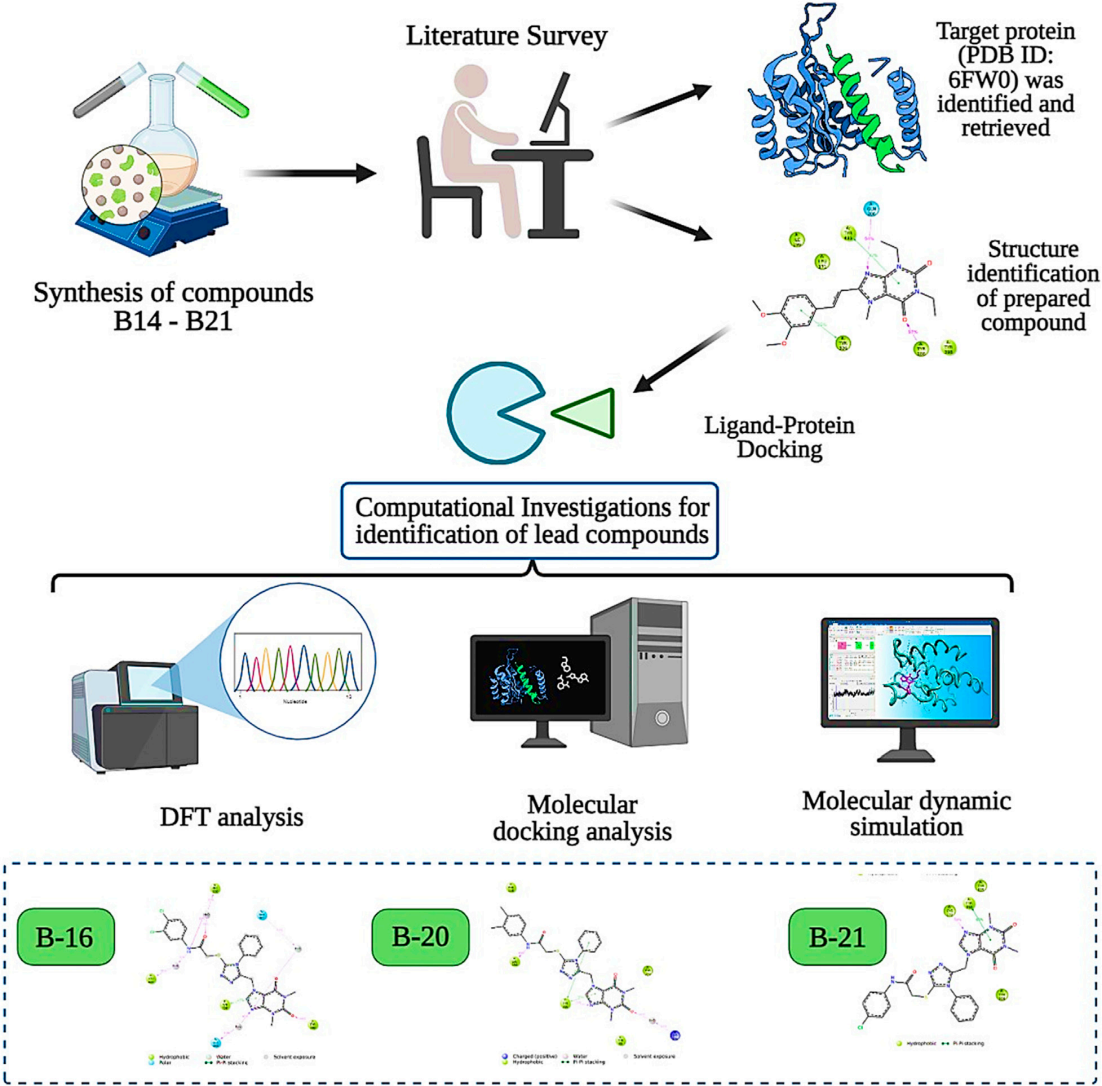
CADD approach workflow of acefylline derivatives as MAO-B inhibitors.

The structure–activity relationship data (presented in Figure 3) encouraged us to carry out *in silico* studies to screen 43 acefylline compounds for MAO-B inhibition activity by utilizing the molecular fragment hybridization and computer-aided drug design (CADD) approaches such as molecular docking, molecular dynamics (MD) simulation, and density functional theory (DFT). The different steps involved in the CADD approach are depicted in the workflow shown in Figure 4.

## 2 Experimental work

### 2.1 Chemistry

The 43 different acefylline moiety-containing compounds were synthesized and reported by our group, as displayed in Supplementary Table S1, which were docked against the MAO-B enzyme protein for the determination of lead MAO-B inhibitors (Shahzadi et al., 2022a; Shahzadi I. et al., 2022; Shahzadi et al., 2020; Shahzadi et al., 2021; Shahzadi et al., 2022c).

### 2.2 Protein structure preparation

The crystal structure of the target protein (PDB ID: 6FW0; resolution = 1.60 Å) (Reis et al., 2018) was obtained from the RCSB Protein Data Bank (https://www.rcsb.org/) (Sobala et al., 2020; Kouranov et al., 2006). The protein structure was prepared by removing water molecules beyond 5 Å of the active site and the co-crystallized ligand, chlorophenyl-chromone-carboxamide (E92). Hydrogen atoms were added, and the protein structure was protonated to ensure proper ionization states of amino acid residues.

### 2.3 Ligand structure preparation

The 3D structures of the ligand molecules were prepared using ChemDraw and optimized using Avogadro software (Mendelsohn, 2004; Hanwell et al., 2012). The valences were satisfied, and charges were fixed, followed by energy minimization using Amber10ff: EHT. The ligand structures were saved in Mol2 file format for docking studies.

### 2.4 Molecular docking

Molecular docking simulations were performed using GOLD software (Verdonk et al., 2003). The active site residues were defined based on the sulfur of Cys172 (derived from previous experimental studies) based on its consistent hydrogen interaction with all the reported MAO-B inhibitors. A cavity was defined around this sulfur atom. The genetic algorithm with a population size of 1,000, the number of runs set to 10, a crossover rate of 95%, and a default mutation rate was selected for docking. The number of genetic algorithm operations was set to 100. The default scoring function implemented in GOLD was used to evaluate the fitness of the docked conformations. The output poses generated by GOLD were analyzed based on binding affinity scores and binding interactions. The top-ranked pose with the highest GOLD fitness score was again subjected to energy minimization using YASARA to remove any steric clashes and unfavorable bumps/interactions (Krieger et al., 2002). The visualization and interpretation of the docking results were performed using molecular visualization software PyMOL Molecular Graphics System (DeLano, 2002). The docking protocol was validated by comparing the docking pose of safinamide with the crystal pose that yielded significant overlap and a root mean square deviation (RMSD) of 1.2 Å (Supplementary Figure S1).

### 2.5 Molecular dynamics simulation

The protein–ligand docked complexes were meticulously prepared using Maestro 12.5 Protein Preparation Wizard (PPW) to correct any structural irregularities, while Prime was used to reconstruct missing side chains and loops. To assess the dynamic behavior and changes in the protein structure in a solvated environment, MD simulations were performed by utilizing the Desmond module (Ali et al., 2024). The Desmond System Builder was used to construct the solvated system, placing the complex in an orthorhombic cubic box with periodic boundary conditions, following previous studies (Zahid et al., 2023; Ali et al., 2023; Bowers et al., 2006). The simulation box was then thoroughly filled with single-point charge (SPC) water molecules, which maintained a minimum distance of 10 Å between the box boundaries and any protein atom (Wu et al., 2006). To achieve charge neutrality within the system, the counterions (Na+ and Cl−) were randomly introduced in the appropriate number. Additionally, 0.15 M NaCl was added to achieve isotonic conditions. As the system was fully solvated, it underwent a series of energy minimization and relaxation steps, following the standard procedures outlined in Desmond’s default protocol, utilizing the OPLS3e force field parameters (Roos et al., 2019). The simulation was carried out at a constant temperature of 300 K and pressure of 1 atm, maintained by the Nose–Hoover thermostat and Martyna–Tobias–Klein barostat algorithms (Boulaamane et al., 2023b). A 100-ns simulation was performed, with 1,000 trajectories saved throughout the run. The Simulation Interaction Diagram (SID) tool was used for the analysis of the resulting MD simulation trajectory for detailed insights into the structural changes and stability of the protein–ligand complex over time (Boulaamane et al., 2023c).

### 2.6 Binding free energy analysis

The binding free energies of the selected docked complexes were revealed by molecular mechanics energies combined with the generalized Born and surface area solvation (MM/GBSA) method (Wang et al., 2019). For this, 5,000 simulation snapshots were picked from the simulation trajectories and analyzed with the equation given below:

$$G=E\;\_\left(\text{bnd}\right)+E\;\_\left(\text{el}\right)+E\;\_\left(\text{vdW}\right)+G\_\left(\text{pol}\right)+G\_\left(\text{np}\right)\;-\text{TS},$$

where E_bnd_, E_el_, E_vdW_, G_pol_, G_np_, and TS stand for bonding energy, electrostatic energy, van der Waals energy, polar solvation energy, and absolute temperature multiplied by entropy energy, respectively. The details of the above equation are given in the study by Genheden and Ryde (2015).

### 2.7 Density functional theory studies of acefylline

Density functional theory (DFT) studies were conducted on the most active compounds (**MAO-B14**, **MAO-B15**, **MAO-B16**, **MAO-B20**, and **MAO-B21)** to gain insights into their electronic properties and the stability of the orbitals involved. These studies were carried out according to previously reported studies using the Gaussian program (Dennington et al., 2008; Muhammed and Aki-Yalcin, 2023). The calculations of the program provided critical parameters such as the total energy, the highest occupied molecular orbital (HOMO) energy, and the lowest unoccupied molecular orbital (LUMO) energy for each acefylline compound. These values were used to derive parameters such as the energy gap between the HOMO and LUMO orbitals, which indicated the compounds’ chemical reactivity and stability. In the context of understanding the observed biological activities, the DFT results provided theoretical foundations on the basis of the electronic structures of the compounds.

### 2.8 Computational pharmacokinetic study

A computational pharmacokinetic study was conducted for the assessment of the absorption, distribution, metabolism, excretion, and toxicity (ADMET) properties of the compounds, which displayed relatively higher inhibition potency against the MAO-B enzyme of PD. The key parameters atomic logarithmic partition coefficient (AlogP), polar surface area-2 dimensional (PSA-2D), BBB permeability level, and Ames mutagenicity of the compounds were calculated using Discovery Studio Client 3.5. Furthermore, the acefylline compounds were evaluated for their compliance with the RO5 (Lipinski’s rule of five), which predicts the bioavailability score, and BBB permeability was calculated through the SwissADME server (http://www.swissadme.ch/) (Daina et al., 2017; Daina and Zoete, 2016). The capability of the compounds to cross the BBB was computed by the two methods and compared afterward (Muhammed et al., 2023).

## 3 Results and discussion

### 3.1 Molecular docking of acefylline derivatives

From molecular dockings, **MAO-B21** emerged with the highest GOLD fitness score of 75.22 followed, by **istradefylline** with 74.91, **safinamide** (73.73), **MAO-B20** (71.94), **MAO-B14** (70.68), **MAO-B15** (68.81), and **MAO-B16** with a docking score of 66.12 (Table 1).

**TABLE 1:** GOLD fitness scores of the most bioactive acefylline derivatives.

Ligand name and structure	GOLD fitness scores
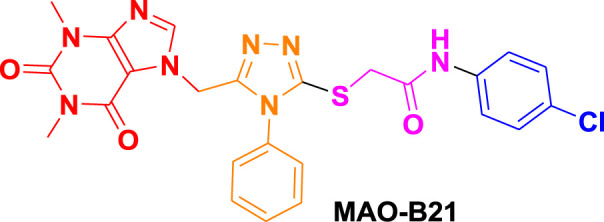	75.22
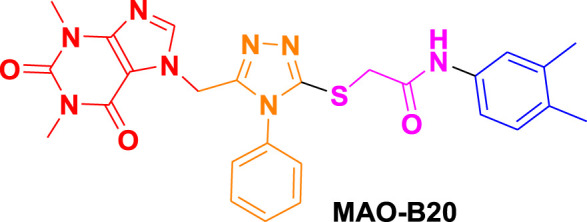	71.94
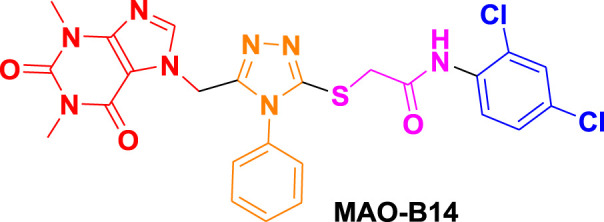	70.68
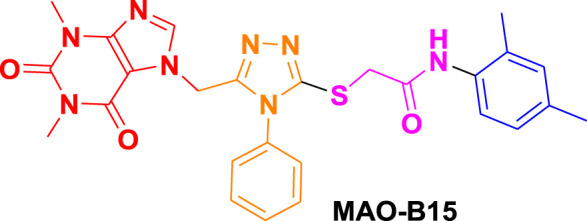	68.81
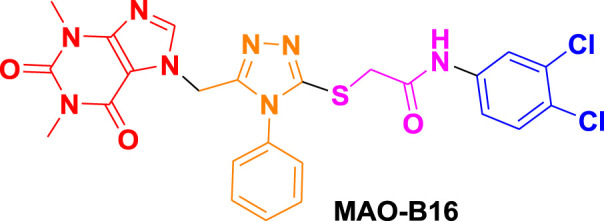	66.12
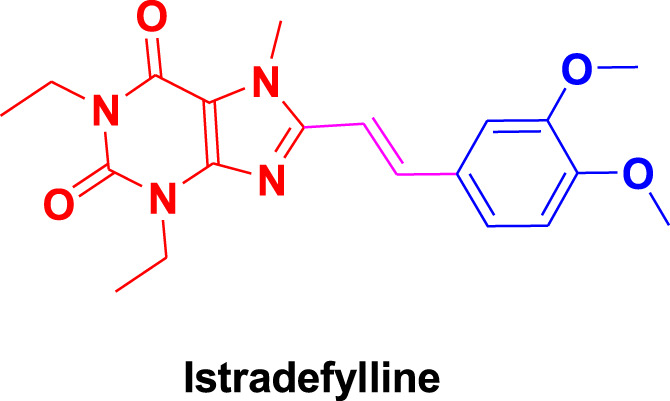	74.91
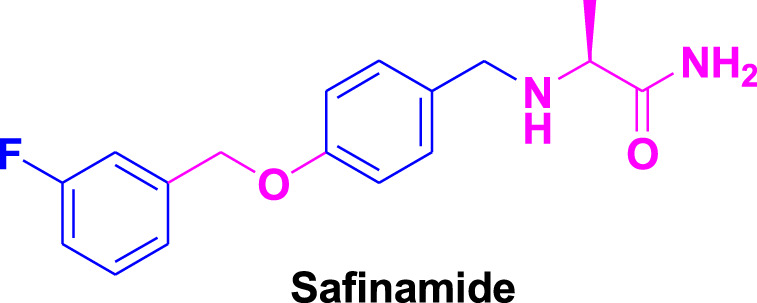	73.73

This high score suggests strong binding affinities of all the compounds, specifically **MAO-B21**, **MAO-B20,** and **MAO-B14** as compared to the control **istradefylline** and **safinamide**.

**FIGURE 5:**
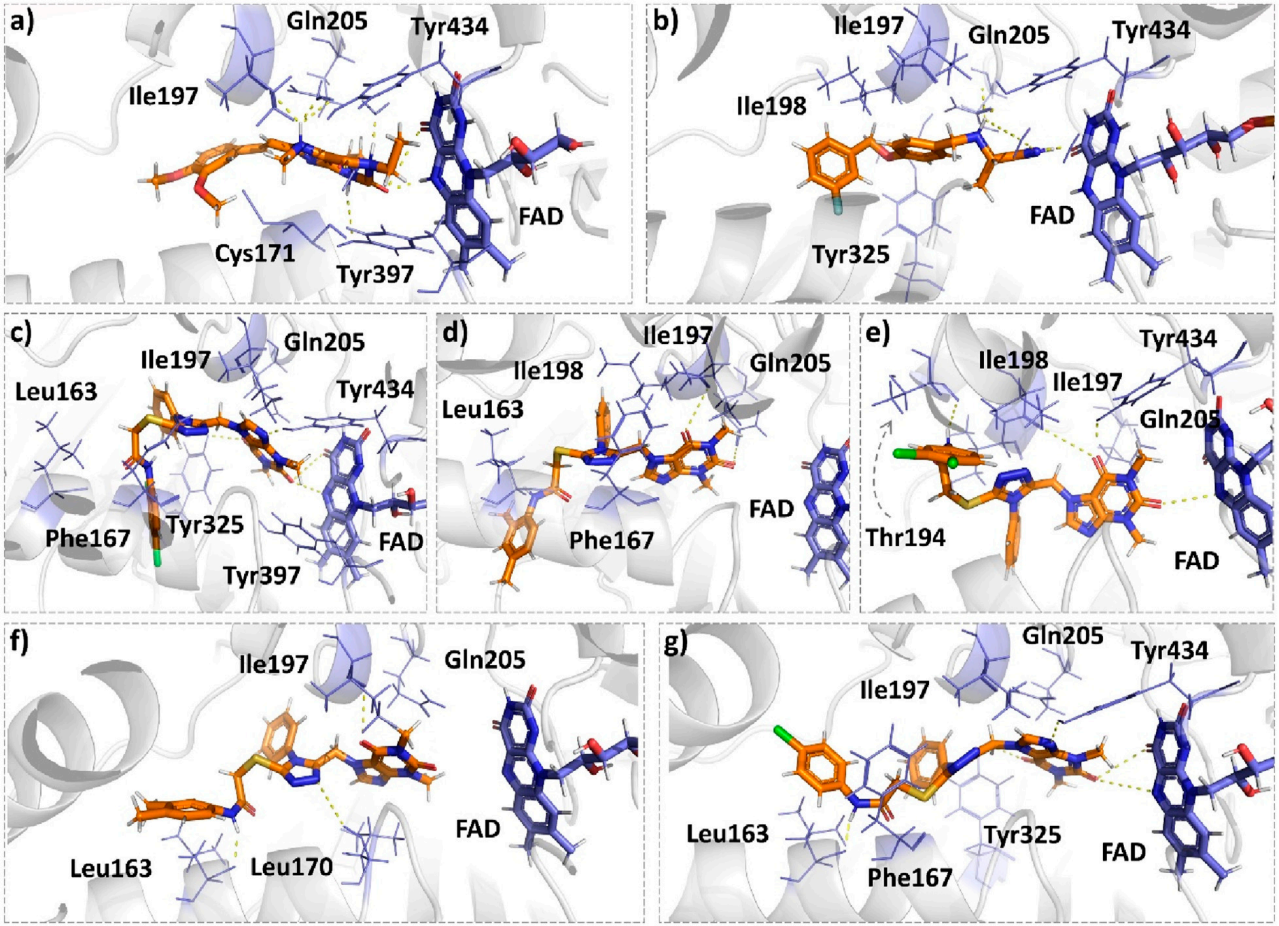
Molecular docking comparison of the control compounds, i.e., **(A)** istradefylline and **(B)** safinamide, with the top five compounds, namely, **(C)** MAO-B14, **(D)** MAO-B15, **(E)** MAO-B16, **(F)** MAO-B20, and **(G)** MAO-B21, against the MAO protein. The hydrogen-bonding interactions are shown in yellow, while the hydrophobic interactions are shown in red. The figure was rendered using PyMOL Molecular Graphics System v2.5.

Istradefylline forms conventional hydrogen bonding interactions with Ile197, Gln205, and Tyr397, and the FAD, enhancing its binding affinity (Figure 5A). Additionally, it engages in hydrogen-bonding interactions with three water molecules within the active site and π–cation interactions with both Tyr397 and FAD (Supplementary Figure S2A). It also establishes notable hydrophobic interactions with amino acids like Pro103, Leu163, Leu166, and Leu170. These hydrophobic interactions reinforce the ligand’s affinity for the active site of the MAO protein. The presence of miscellaneous interactions (Table 2) such as halogen interaction with Leu166 and Phe167 and sulfur interaction with Cys171 contributes to its overall binding profile. Safinamide establishes conventional hydrogen interactions with Tyr325 and Tyr434 and carbon–hydrogen interaction with Phe167 and Ile198, as well as one water hydrogen bond, indicating a considerable number of hydrogen bonds stabilizing the complex (Figure 5B). The ligand also demonstrates hydrophobic interactions, i.e., π–sigma interactions with Ile198, π–π T-shaped interactions with Tyr325, and π–alkyl interactions with Pro103, Leu163, Leu170, and Ile198, further confirming its binding strength. π–sulfur interactions with an important residue Cys171 also contribute to its unique binding profile (Table 2) (Supplementary Figure S2B).

**TABLE 2:** Ligands with all the established hydrogen, hydrophobic, and miscellaneous interactions with atoms involving halogens, lone pairs, or sulfur.

**Ligand**	Hydrogen-bonding interactions	Hydrophobic interactions	Miscellaneous (fluorine = F, chlorine = Cl, and sulfur = S. Lone pair = L)
**Istradefylline**	Phe167, Ile197, Gln205, and Tyr397	Pro103, Leu163, Leu166, Leu170, Trp118, Phe167, Ile198 (2), Ile315, Tyr397, and FAD501	**—**
**Safinamide**	Phe167, Ile198, Tyr325, and Tyr434	Pro103, Leu163, Leu170, Ile198 (2), and Tyr325	Leu166(F), Phe167(F), and Cys171(S)
**MAO-B14**	Ile197, Ile198, Gln205, Tyr325 (3), and Tyr434	Tyr59, Leu166 (2), Leu170 (3), Cys171, Ile198, Ile315, Ala324 (2), Tyr325 (2), Leu327, Phe342, Leu344 (2), Tyr397, Tyr434, and FAD501 (2)	Cys171(3S)
**MAO-B15**	Phe167, Ile197 (2), Ile198 Gln205	His89, Leu166, Leu170 (7) Cys171 (3), Val191, Ile197, Ile198 (4), Ile315 (2), Tyr325 (2), Tyr434, and Phe342(2)	**—**
**MAO-B16**	Ile197, Tyr397, and Tyr434	Met121 (2), Leu170 (3), Cys171 (2), Phe184(2), Leu185, Val188, Val188(2), Ile197 (2), Ile198, Ile315, Tyr325 (2), Phe342, Tyr397 (2), Tyr434, and FAD501	Phe167(S), Cys171(S), and Phe184(Cl)
**MAO-B20**	Leu163, Ile197, and Ile198 (2)	His114 (2), Phe117, Ala160, Leu163 (2), Ala164, Leu170 (3), Cys171 (2), Ile197, Ile198 (2), Ile315, Tyr325, Phe342, Tyr397, and Tyr434	**—**
**MAO-B21**	Leu163, Trp118, Ile197 (2), Tyr325, and Tyr434	Tyr59, Phe102, Pro103, His114, Trp118, Leu163, Leu170, Ile198 (2), Ile315, Tyr325, Leu327, Phe342, Tyr434, FAD501 (2), Leu170, Cys171, and Leu170	Phe167(S) and Cys171(S)

**MAO-B14** forms conventional hydrogen bonds with Gln205, Tyr325, and Tyr434 and carbon–hydrogen bonds with Ile197 and Ile198 (Figure 5C). Sulfur and π–sulfur interactions were found with Cys171. Hydrophobic interactions were observed with amino acids Tyr59, Leu166, Leu170, and others, making it a good binder (Supplementary Figure S2C). **MAO-B15** probably has the worst binding network with no conventional hydrogen bonds but only forms carbon–hydrogen bonds with Phe167, Ile197, Ile198, and Gln205 and numerous hydrophobic interactions (Figure 5D). The multiple interactions with Leu170, Cys171 Tyr325, Ile315, and other amino acids further illustrate its binding potential (Supplementary Figure S2D). **MAO-B16** only establishes conventional hydrogen bonds with Tyr434; carbon–hydrogen interactions with Ile197, Tyr397, and Tyr434 (Figure 5E); and a range of hydrophobic interactions with amino acids Met121, Leu170, Cys171, Phe184, and others. Other miscellaneous interactions, including those of sulfur (S) with Cys171 and Phe167 and chlorine (Cl) with Phe184, contribute to its binding characteristics (Supplementary Figure S2E). **MAO-B20** engages in conventional hydrogen-bonding interactions only with Leu163 and carbon–hydrogen interactions with Ile197 and Ile198 (Figure 5F). It also establishes numerous hydrophobic interactions with His114(2), Phe117, Ala160, Leu163(2), Ala164, Leu170(3), Cys171(2), Ile197, Ile198(2), Ile315, Tyr325, Phe342, Tyr397, and Tyr434. The presence of multiple interactions underscores its binding affinity (Supplementary Figure S2F). **MAO-B21** exhibits multiple conventional hydrogen-bonding interactions with Leu163, Tyr325, and Tyr434 and carbon–hydrogen-bonding interactions with Trp118 and Ile197 (Figure 5G). These hydrogen bonds play a critical role in stabilizing the ligand–protein complex. It also establishes π–sulfur bonds with Phe167 and the important active site residue Cys171. Additionally, it engages in numerous hydrophobic interactions with amino acids like Tyr59, Phe102, Pro103, His114, and Trp118 (Supplementary Figure S2G). These hydrophobic interactions indicate the strength of nonpolar forces contributing to the binding. These molecular docking results reveal the diverse binding profiles of the top compounds. **MAO-B21**, **istradefylline**, **safinamide**, **MAO-B20,** and **MAO-B14** demonstrate a strong binding affinity with a substantial number of interactions, particularly hydrogen and hydrophobic interactions, indicating their potential as MAO protein binders. The remaining ligands also exhibit varying degrees of interactions, contributing to their binding with the MAO protein.

### 3.2 Molecular dynamics analysis of MAO-B inhibitors

The molecular docking study results can be corroborated through MD simulations, providing a dynamic assessment of the stability of the chosen NPs. Various parameters, such as the RMSD of Ca atoms, ligand RMSD relative to the protein, root-mean square fluctuation (RMSF) of C-alpha atoms in the proteins, and protein–ligand interactions, were scrutinized from the trajectories of the MD simulations.

#### 3.2.1 Root mean square deviation analysis of MAO-B inhibitors

**FIGURE 6:**
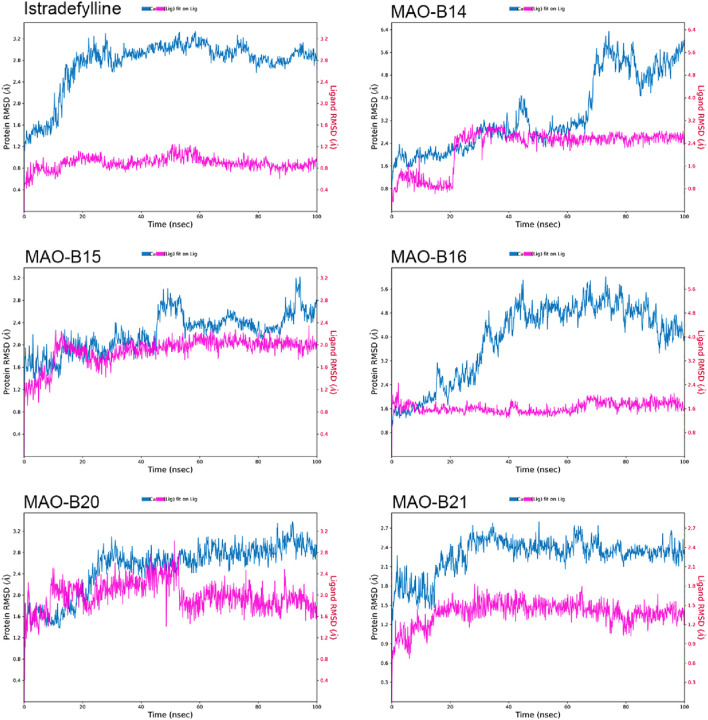
RMSD plots of the selected ligands in complex with MAO-B over a 100-ns simulation.

The RMSD plots of the protein and ligand were generated using 100-ns molecular dynamics simulations to demonstrate the temporal stability of the protein–ligand complexes (Figure 6). The RMSD is calculated for the atomic positions of the protein and ligand relative to their initial positions, with lower RMSD values indicating greater stability of the protein–ligand complex.

The reference complex, MAO-B–istradefylline, exhibits an initial increase in protein RMSD before stabilizing at approximately 3.0–3.2 Å after approximately 20 ns. The istradefylline ligand shows minimal fluctuations, maintaining a stable RMSD of approximately 0.8–1.0 Å throughout the simulation. This pattern suggests a stable binding mode for the reference compound.

Among the studied acefylline derivatives, **MAO-B14** and **MAO-B16** demonstrated the most significant protein RMSD fluctuations. The **MAO-B14** protein RMSD increases steadily, reaching up to 6 Å by the end of the simulation, while its ligand RMSD also shows a gradual increase from approximately 1 Å to 2.5 Å. MAO-B16 exhibited large protein RMSD fluctuations between 3 and 5 Å, although its ligand RMSD remained relatively stable at approximately 1.5–2.0 Å.

**MAO-B15** and **MAO-B20** displayed protein RMSD patterns more similar to those of istradefylline, with **MAO-B15** stabilizing at approximately 2.5 Å and **MAO-B20** showing slightly higher fluctuations between 2.5 and 3.0 Å. Their ligand RMSDs were marginally higher than those of istradefylline, with the MAO-B15 ligand fluctuating at approximately 2.0 Å and **MAO-B20** showing more variation between 1.5 and 2.5 Å.

Notably, the **MAO-B21** complex exhibited the most stable protein RMSD trend among the acefylline derivatives, reaching a plateau at approximately 2.4 Å after an initial adjustment period. Its ligand RMSD also remained consistently low, approximately 1.5 Å, indicating a stable binding mode comparable to that of istradefylline. These RMSD analyses provide insights into the relative stabilities of the complexes over time, complementing the molecular docking studies and highlighting promising candidates for further investigation based on their stability profiles.

#### 3.2.2 RMSF analysis

**FIGURE 7:**
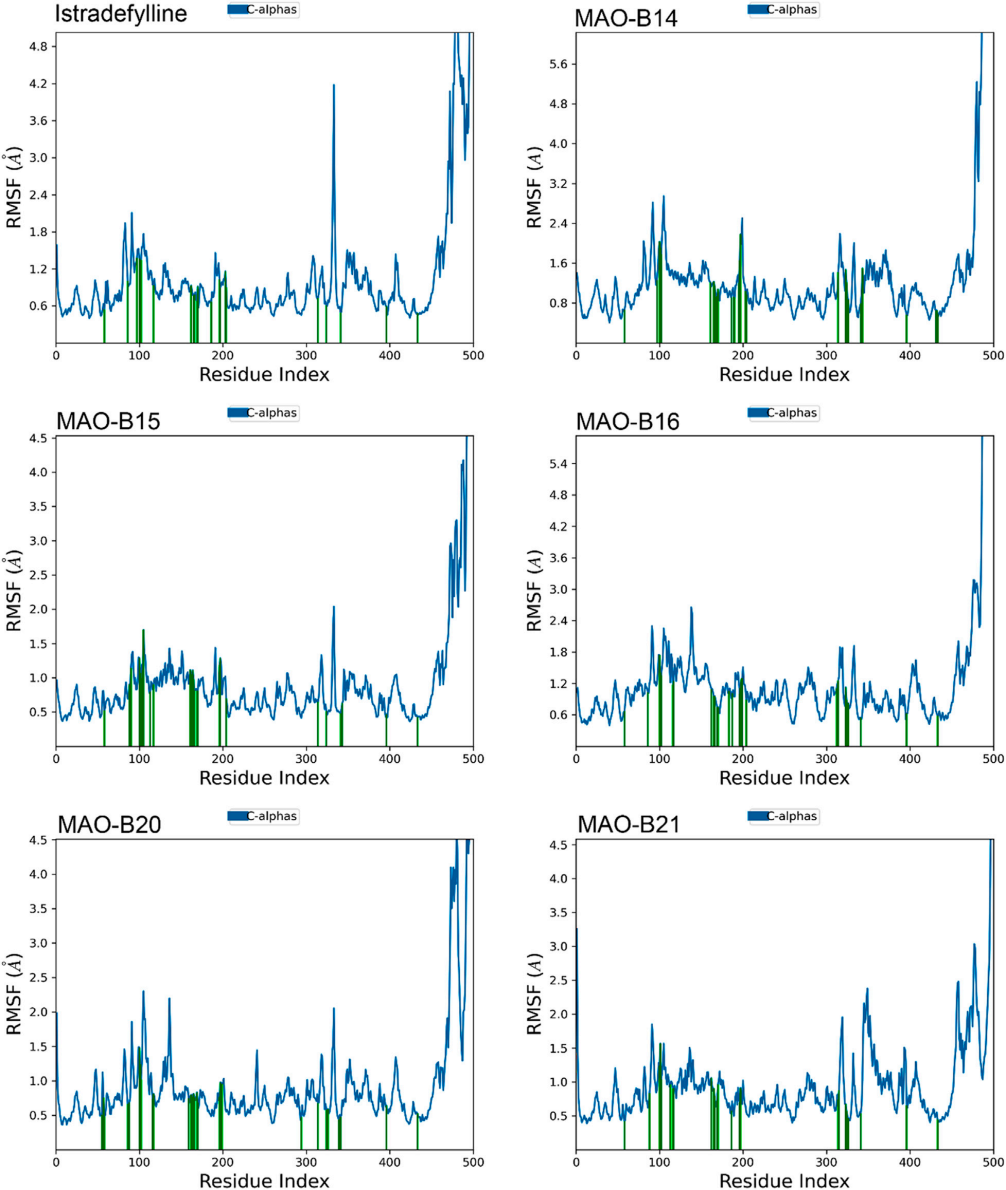
RMSF plots of the chosen ligands interacting with MAO-B illustrate the fluctuation in individual amino acids over the duration of the simulation.

The RMSF analysis of the MD simulations provided valuable insights into the dynamic behavior of the MAO-B protein complexes. The RMSF plots revealed distinct patterns of fluctuations across different regions of the protein structure (Figure 7).

One notable observation is the presence of substantial fluctuations (4–5+ Å) in the C-terminal region, at approximately residue 500. This heightened mobility was consistent with the non-binding role and inherent flexibility of this region. In contrast, the majority of the protein structure exhibited lower fluctuations (1–2 Å), indicating overall stability and rigidity.

However, all complexes demonstrated slightly elevated fluctuations (2–3 Å) at approximately residues 100–110 and 200–210, suggesting potential flexibility or conformational changes in these specific regions. Interestingly, alpha-helical regions, represented by vertical green lines, generally exhibited lower fluctuations, which are expected due to their structural stability (Figure 7).

Although the overall RMSF profiles appeared similar across complexes, subtle differences can be observed. For instance, MAO-B16 displayed slightly higher overall fluctuations, while MAO-B21 exhibited lower fluctuations in the 300–400 residue range. These minor variations may indicate subtle conformational changes induced by the binding of different ligands (Figure 7).

Notably, the binding site residues are depicted by vertical green lines, which typically exhibit lower fluctuations. This observation suggests that the potential ligand-binding areas remained relatively stable during the simulations. However, a comprehensive analysis of the specific binding site interactions would require additional information, such as the three-dimensional structures and docking poses of the bound ligands.

#### 3.2.3 Protein–ligand interactions

**FIGURE 8:**
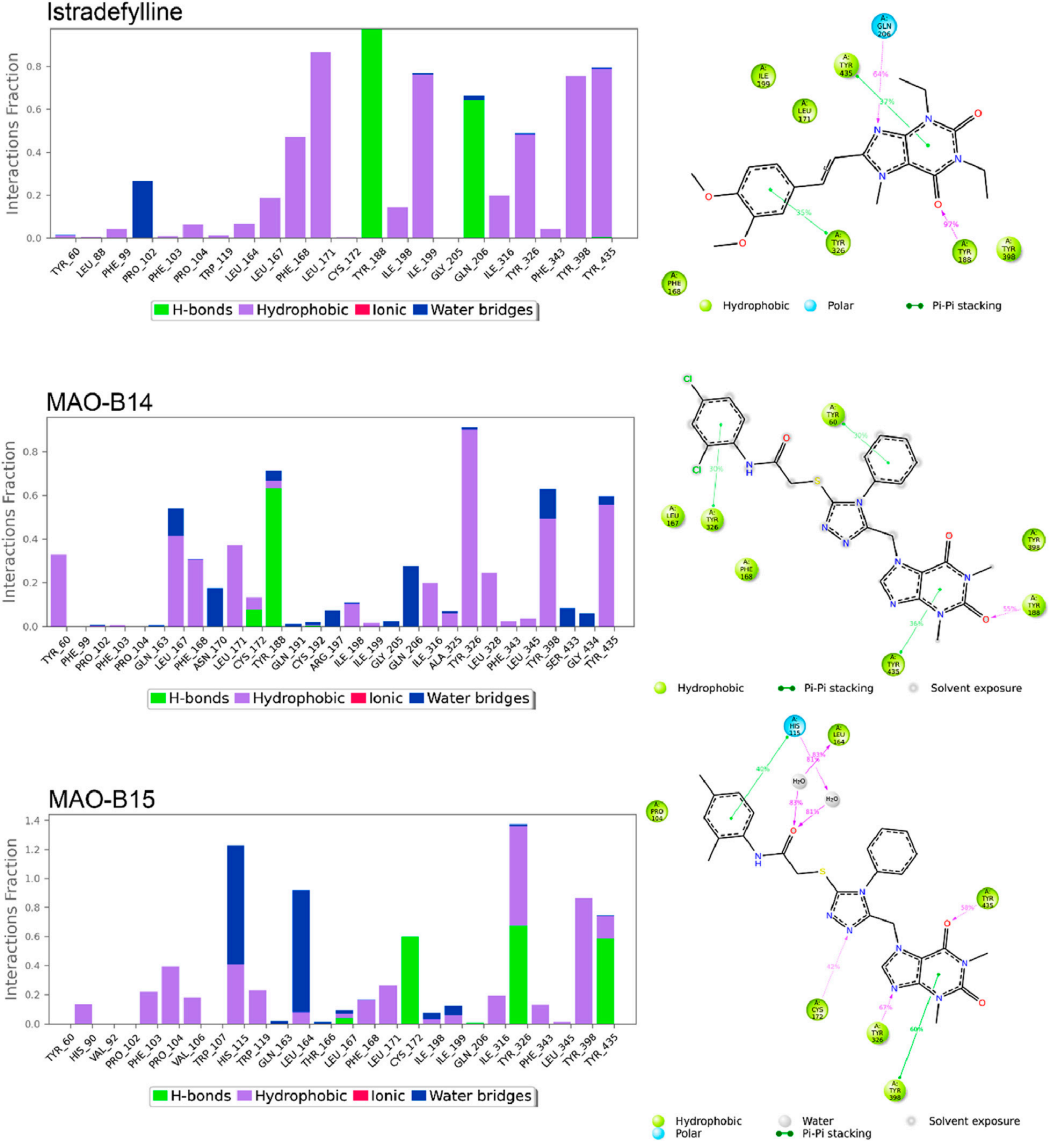
Simulation interactions of istradefylline, MAO-B14, and MAO-B15. The 2D binding interaction along with bar diagram indicating the fold of interaction, fraction, and contacts are also shown on the left.

**FIGURE 9:**
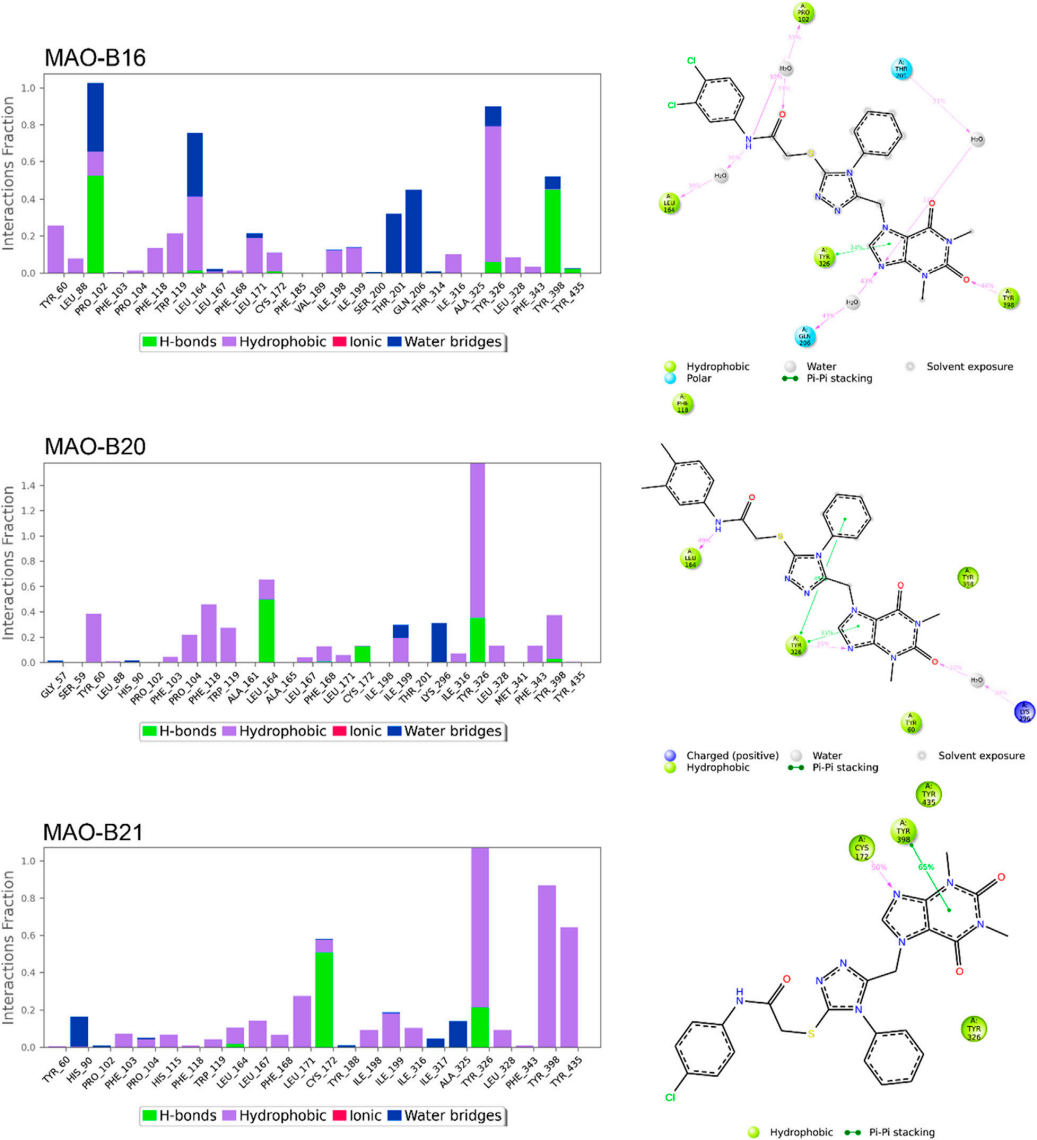
Simulation interactions of MAO-B16, MAO-B20, and MAO-B21. The 2D binding interaction along with bar diagram indicating the fold of interaction, fraction, and contacts are also shown on the left.

Figures 8, 9 show the various protein–ligand bonds, along with their respective interaction fractions for the examined complexes. The results highlighted the prevalence of hydrophobic interactions involving Leu-171, Ile-198, Ile-199; Tyr-326, Tyr-398, and Tyr-435, which collectively form an aromatic cage. Notably, the reference complex exhibited key hydrogen bonds with Tyr-188 and Gln-206.

In the case of **MAO-B14**, the interaction profile closely corresponded to that of istradefylline. However, a distinctive feature involved the presence of a significant hydrogen bond with Tyr-188. Conversely, **MAO-B15** showcased the formation of new hydrogen bonds with Cys-172, Tyr-326, and Tyr-435.

**MAO-B16** established a hydrogen bond with Pro-102 near the entrance cavity, indicating that the ligands occupy both cavities in MAO-B. This could potentially signify favorable selectivity. Moreover, the acefylline moiety, directed toward the FAD, formed two hydrogen bonds with Tyr-398 and Tyr-435.

In the case of **MAO-B20**, a consistent interaction was observed with Tyr-326. Finally, **MAO-B21**, akin to **MAO-B15**, formed a crucial hydrogen bond with Cys-172, which plays a pivotal role in stabilizing and anchoring MAO-B inhibitors.

#### 3.2.4 Hydrogen bonds and pi–pi stacking interactions

**FIGURE 10:**
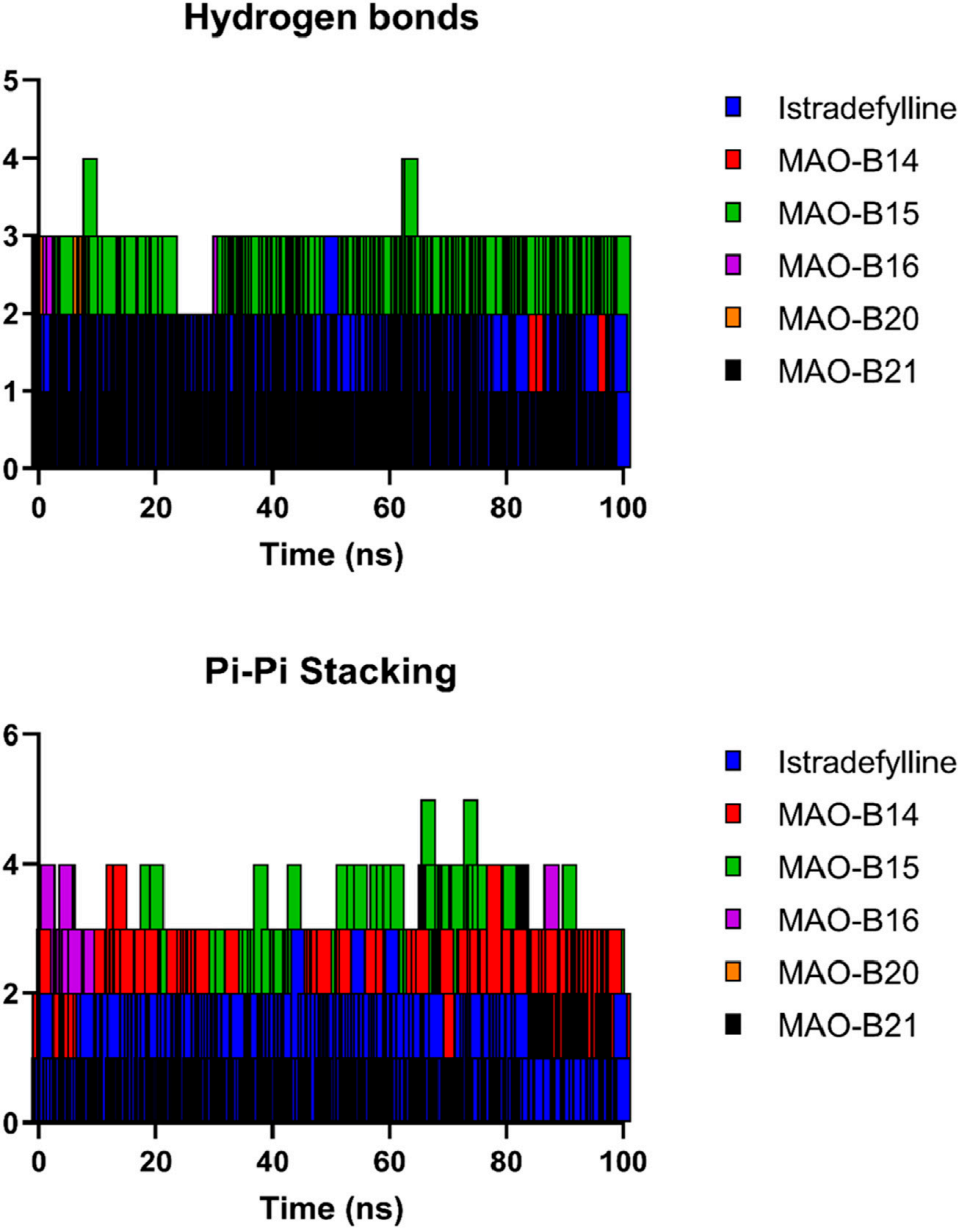
Hydrogen bonds and pi–pi stacking interactions of the selected complexes throughout the simulation time.

Figure 10 depicts the fluctuation in the count of hydrogen bonds across the five protein–ligand complexes throughout the simulation. These hydrogen bonds serve as indicators of the interaction strength between the protein and the ligand, with a higher count reflecting a more robust interaction. Over the course of the simulation, the number of hydrogen bonds in all five complexes exhibited fluctuations, indicating overall stability in the protein–ligand complexes during the simulation period. Variations in the number of hydrogen bonds among the complexes suggested differing affinities of the ligands for the proteins. Notably, the **MAO-B15** complex consistently maintained the highest number of hydrogen bonds, implying its strong affinity for the protein. Conversely, the **MAO-B20** complex demonstrated the lowest count of hydrogen bonds, indicating its weaker affinity for the protein. Meanwhile, the istradefylline, **MAO-B14**, and **MAO-B16** complexes exhibited similar numbers of hydrogen bonds, suggesting comparable affinities for the protein. The slight fluctuations observed in the hydrogen bond counts across all complexes over time are likely attributed to the thermal motion. Additionally, the pi–pi stacking plot further emphasized the potential of **MAO-B15** as a promising lead candidate for MAO-B inhibition as it demonstrated the highest number of aromatic interactions, crucial to ligand binding.

#### 3.2.5 Binding free energy estimation using the MM-GBSA approach

Molecular docking strategy-based binding energy values of the generated complexes often face high chances of false-positive results. Therefore, simulation-based binding free energies *via* the MM-GBSA method were predicted. As per the results given in Table 3, the compounds showed robust van der Waals energy in complex with the receptor enzyme. Furthermore, the compounds showed that electrostatic energy plays a favorable role in the intermolecular complex. In contrast, the solvation energy of the complexes witnessed a negative contribution to complex formation. The net MM-GBSA binding energy of **istradefylline**, **MAO-B14**, **MAO-B15**, **MAO-B16**, **MAO-B20,** and **MAO-B21** is −77.49 kcal/mol, −76.5 kcal/mol, −108.84 kcal/mol, −69.23 kcal/mol, −64.98 kcal/mol, and −81.29 kcal/mol, respectively.

**TABLE 3:** MM-GBSA-based binding free energies. The values are presented in kcal/mol.

Parameter	Istradefylline	MAO-B14	MAO-B15	MAO-B16	MAO-B20	MAO-B21
Van der Waals energy term	−66.84	−68.25	−71.01	−65.24	−60.34	−70.11
Electrostatic energy term	−25.34	−24.12	−27.10	−16.33	−18.74	−26.38
Gas-phase energy term	−92.18	−92.37	−125.21	−81.57	−79.08	−96.49
Solvation energy term	14.69	15.87	16.37	12.34	14.10	15.20
Net energy term	−77.49	−76.5	−108.84	−69.23	−64.98	−81.29

### 3.3 DFT results

The DFT computation approach was conducted to determine the total, highest occupied molecular orbital (HOMO), and least occupied molecular orbital (LUMO) energies of the relatively active compounds among the investigated acefylline derivatives. Based on the values obtained, the other electrochemical parameters were computed with the appropriate theorems. The ionization potential (*IP* = -*E*_HOMO_), electron affinity (*A* = -*E*_LUMO_), hardness (*η* = (*I*-*A*)/2), softness (*S* = 1/2*η*), Mulliken electronegativity ( X = (*I* + *A*)/2) (Parr et al., 1978), electrophilicity index (*ω* = *µ* ^2^/2*η*) (Chattaraj et al., 2006), chemical potential (*µ* = -(*I* + *A*)/2), and maximum charge transfer (*ΔN*_max_ = (*I* + *A*)/2(*I*-*A*)) were computed (Table 4) (Koopmans, 1934).

**TABLE 4:** Molecular orbital energy and related parameters of the relatively active acefylline derivatives (in eV).

Parameter	MAO-B14	MAO-B15	MAO-B16	MAO-B20	MAO-B21
***E***_**total**_	−44,350.9	−45,419.1	−44,351.8	−45,710.1	−43,962.0
***E***_**HOMO**_	−6.065	−8.681	−6.185	−6.006	−6.182
***E***_**LUMO**_	−1.741	−1.808	−1.654	−1.906	−1.707
***ΔE***	4.324	6.873	4.531	4.100	4.475
***IP***	6.065	8.681	6.185	6.006	6.182
***A***	1.741	1.808	1.654	1.906	1.707
***µ***	−3.903	−5.245	−3.920	−3.956	−3.945
***Η***	2.162	3.437	2.266	2.050	2.238
X	3.903	5.245	3.920	3.956	3.945
***S***	0.231	0.145	0.221	0.244	0.223
***ω***	3.519	3.989	3.396	3.819	3.471
***ΔN***_**max**_	0.902	0.763	0.865	0.965	0.881

The DFT studies showed that there was similarity among some derivatives and difference among some others in terms of the values obtained. HOMO and LUMO energy values were utilized to compare the electron exchange ability of the active compounds. Compound **MAO-B20** yielded the highest HOMO energy value (Table 4). As HOMO energy represents the ability to donate electrons, **MAO-B20** is anticipated to possess the highest electron-donating capability (Akman et al., 2023). Compound **MAO-B16** yielded the highest LUMO energy value (Table 4). Hence, it is expected to have the highest tendency to accept electrons readily as LUMO represents the ability to do so (Miar et al., 2021). The LUMO–HOMO energy gap is important in measuring the relative stability of compounds. Compounds with a higher energy gap generally have a higher chemical stability (Ruiz-Morales, 2002). In the DFT study, **MAO-B15** yielded the highest energy gap among the active compounds (Table 4). Hence, compound **MAO-B15** exhibits the highest chemical stability. Global hardness represents the atoms’ resistance to transfer electrons. In the DFT study, **MAO-B15** gave the highest global hardness value (Table 4). To summarize, compound **MAO-B15** has the highest chemical stability and the least reactivity according to the DFT studies (Han et al., 2022). On the other hand, global softness depicts the propensity of a compound for reactivity. Among the investigated compounds, **MAO-B20** had the highest softness value, so **MAO-B20** is anticipated to show the highest reactivity.

**FIGURE 11:**
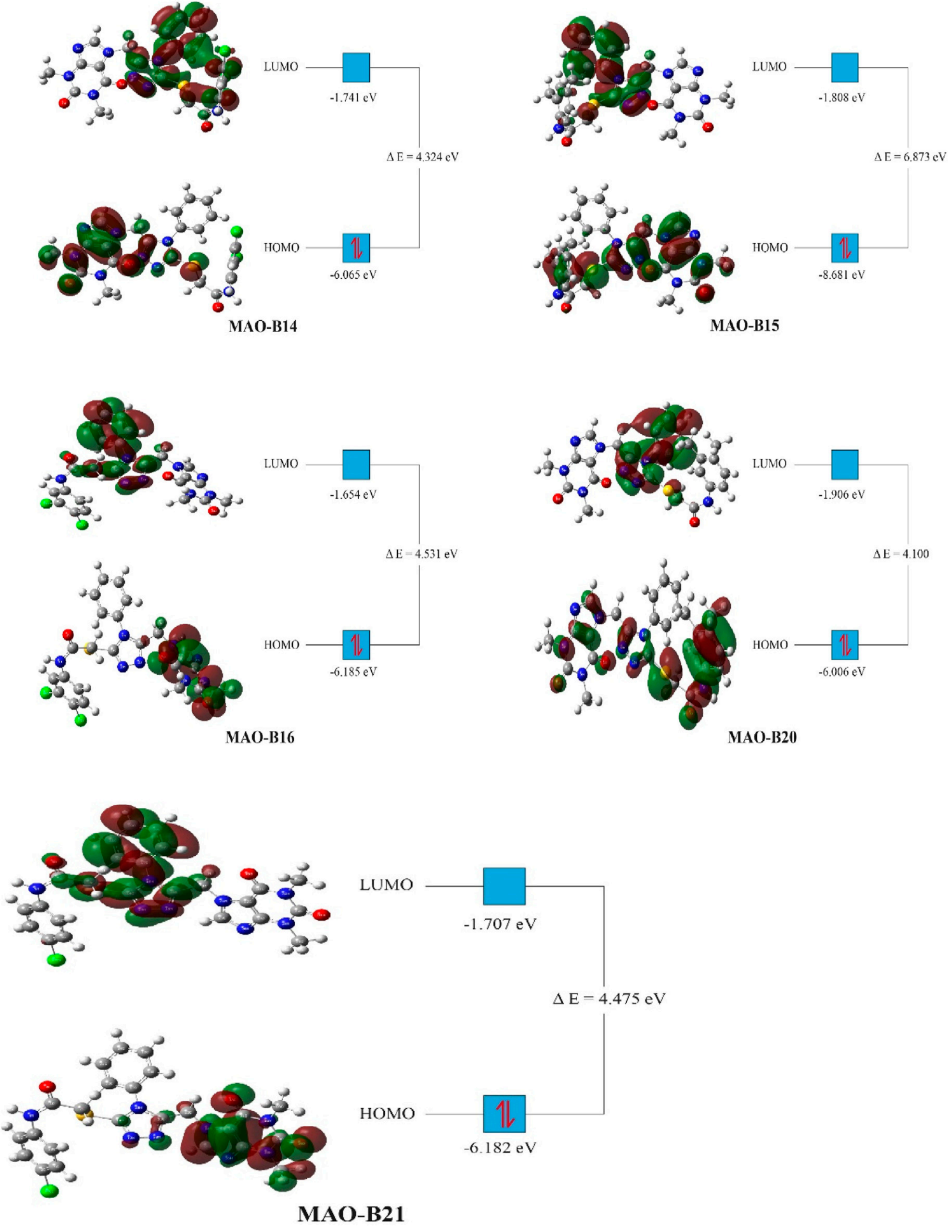
Molecular orbital distribution of the active compounds.

The distribution of HOMOs in the active compounds had similarity in some vicinities and difference in some others. All the investigated compounds had a high concentration of HOMOs on the purine group. Together with this, compounds **MAO-B14**, **MAO-B15**, and **MAO-B20** had molecular orbital concentrations around the triazole group and acetamide functional group. In addition, compounds **MAO-B15** and **MAO-B20** had molecular orbital concentrations around the phenyl group as well (Figure 11). The LUMO distribution of the compounds was similar to each other with small differences. The LUMOs were mainly concentrated around the triazole group, the phenyl ring substituted on it and, to some extent, the acetamide functional group. Compound **MAO-B14** also had molecular orbital concentrations around the phenyl group connected to the functional group (Figure 11).

### 3.4 Computational pharmacokinetic study

The ADMET properties and drug-likeness of the compounds were calculated and interpreted accordingly. All of the investigated compounds infringed two parameters of the RO5, as displayed in Table 5. Lipinski’s rule allows the violation of just a clause for a compound to be compliant to the rule (Lipinski et al., 2001). The computation results implied that the compounds might not be obeyed by the RO5. Hence, the compounds are expected not to be suitable for oral administration. The computation showed that the molecular mass of the compounds exceeded the limit (500 Da) and bore greater than 10 hydrogen bond acceptors (oxygen and nitrogen in this case). The computation implied the necessity to consider these properties in future modifications on the compounds to make them compliant to the RO5 and, thus, exhibit drug-like properties.

**TABLE 5:** ADMET properties and drug-likeness of the most biologically potent derivatives.

Compound	AlogP98	PSA-2D	Bioavailability score	BBB level	Ames mutagenicity	RO5 violations
**MAO-B14**	4.396	115.897	0.17	4	Non-mutagen	2
**MAO-B15**	4.040	115.897	0.17	4	Non-mutagen	2
**MAO-B16**	4.396	115.897	0.17	4	Non-mutagen	2
**MAO-B20**	4.040	115.897	0.17	4	Non-mutagen	2
**MAO-B21**	3.732	115.897	0.17	4	Non-mutagen	2

The case of the compounds to cross the BBB was computed. The program predicted that the BBB would not be permeant to the compounds (Table 5). Similarly, the BBB is estimated to be non-permeant to the compounds according to the SwissADME server estimation. The high number of atoms with a high electronegativity property is expected to decrease the compound’s capacity to cross the BBB. Hence, decreasing the number of hydrogen bond acceptors is expected to contribute to fixing this issue. On the other hand, all the compounds were ascertained to be non-mutagenic (Table 5).

**FIGURE 12:**
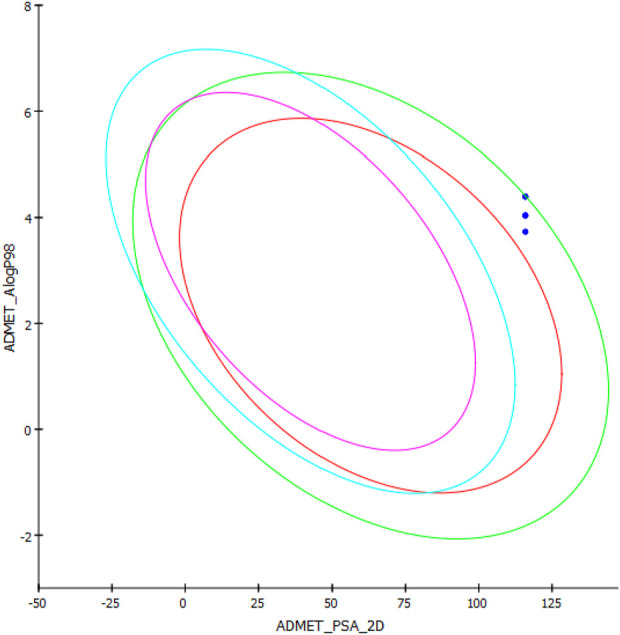
AlogP98 *versus* PSA-2D plot of the compounds.

AlogP98 was used to evaluate the lipophilic property, and a value of below 5 means an ideal lipophilic property for a compound. All of the compounds gave an AlogP98 value of below 5 (Table 5; Figure 12). PSA-2D is used to evaluate the oral absorption of a compound, and a value of below 100 Å^2^ means an ideal oral absorption. All of the compounds gave a PSA-2D value of above 100 Å^2^ (Table 5; Figure 12). Hence, the compounds were found to possess a non-ideal oral absorption property (Boulaamane et al., 2023b). The polar surface area is correlated to the number of polar atoms of a compound. Hence, one of the measures to alleviate this hurdle is decreasing the number of electronegative atoms.

## 4 Conclusion

The current study evaluated the chemotherapeutic potential of 43 acefylline derivatives as MAO-B inhibitors. Five compounds featuring the acefylline scaffold were custom-designed. DFT studies were carried out to optimize their three-dimensional structures. Molecular docking studies with MAO-B unveiled the superior binding affinity of one specific compound, i.e., **MAO-B21**, in comparison to the standard reference ligands, safinamide and istradefylline. The results from MD simulations corroborated the binding scores achieved from the molecular docking approach, demonstrating the exceptional stability of **MAO-B21**. This was evidenced by minimal deviations in the protein backbone and low fluctuations within the binding site, indicative of strong binding. The molecular interaction analysis further highlighted the significance of Tyr-326 in aromatic interactions, along with the presence of a critical hydrogen bond with Cys-172, essential for anchoring MAO-B inhibitors. The outcomes of this study highlight the therapeutic efficacy of **MAO-B21** as a potential MAO-B inhibitor. Nonetheless, experimental validation through enzymatic assays is imperative to confirm these outcomes in future studies.

## Data Availability

The original contributions presented in the study are included in the article/Supplementary Material; further inquiries can be directed to the corresponding authors.
