# Supplementary material for: Computational exploration of acefylline derivatives as MAO-B inhibitors for Parkinson’s disease: insights from molecular docking, DFT, ADMET, and molecular dynamics approaches
Source: Front Chem. 2024 Oct 8;12:1449165. doi: 10.3389/fchem.2024.1449165 (PMC11493617; doi:10.3389/fchem.2024.1449165)
Supplement: Supplementary file 1 [file DataSheet1.docx]

**Computational Exploration of Acefylline Derivatives as MAO-B Inhibitors for Parkinson’s disease: Insights from Molecular Docking, DFT, ADMET, and Molecular Dynamics Approaches**

Ali Irfan^1^, Ameer Fawad Zahoor^1^*, Yassir Boulaamane^2^, Sadia Javed^3^, Huma Hameed^4^, Amal Maurady^2^, Muhammed Tilahun Muhammed^5^, Sajjad Ahmad^6,7,8^, Aamal A. Al-Mutairi^9^, Irum Shahzadi^1^, Sami A. Al-Hussain^9^, Magdi E. A. Zaki^9^**

**Table 1:** Structures of 43 Acefylline Derivatives

|  |  |
| --- | --- |
|  |  |
|  |  |
|  |  |
|  |  |
|  |  |
|  |  |
|  |  |
|  |  |
|  |  |
|  |  |
|  |  |
|  |  |
|  |  |
|  |  |
|  |  |
|  |  |
|  |  |
|  |  |
|  |  |
|  |  |
|  | |

**Table 2:** Gold fitness Scores of 43 Acefylline Derivatives

| **Acefylline Compounds** | **Gold fitness**  **Score** | **Acefylline**  **Compounds** | **Gold fitness**  **Score** |
| --- | --- | --- | --- |
| MAO-B1 | 35.74 | MAO-B23 | 49.25 |
| MAO-B2 | 42.19 | MAO-B24 | 60.55 |
| MAO-B3 | 42.06 | MAO-B25 | 56.56 |
| MAO-B4 | 38.22 | MAO-B26 | 60.33 |
| MAO-B5 | 39.5 | MAO-B27 | 52.64 |
| MAO-B6 | 38.96 | MAO-B28 | 52.22 |
| MAO-B7 | 33.21 | MAO-B29 | 61.0 |
| MAO-B8 | 56.8 | MAO-B30 | 47.29 |
| MAO-B9 | 55.01 | MAO-B31 | 52.97 |
| MAO-B10 | 52.49 | MAO-B32 | 50.15 |
| MAO-B11 | 55.75 | MAO-B33 | 56.26 |
| MAO-B12 | 58.92 | MAO-B34 | 44.06 |
| MAO-B13 | 52.95 | MAO-B35 | 46.54 |
| **MAO-B14** | **70.68** | MAO-B36 | 55.15 |
| **MAO-B15** | **68.81** | MAO-B37 | 43.31 |
| **MAO-B16** | **66.12** | MAO-B38 | 58.39 |
| MAO-B17 | 63.97 | MAO-B39 | 52.53 |
| MAO-B18 | 59.61 | MAO-B40 | 56.02 |
| MAO-B19 | 56.31 | MAO-B41 | 46.44 |
| **MAO-B20** | **71.94** | MAO-B42 | 51.62 |
| **MAO-B21** | **75.22** | MAO-B43 | 45.07 |
| MAO-B22 | 64.29 |  |  |

**
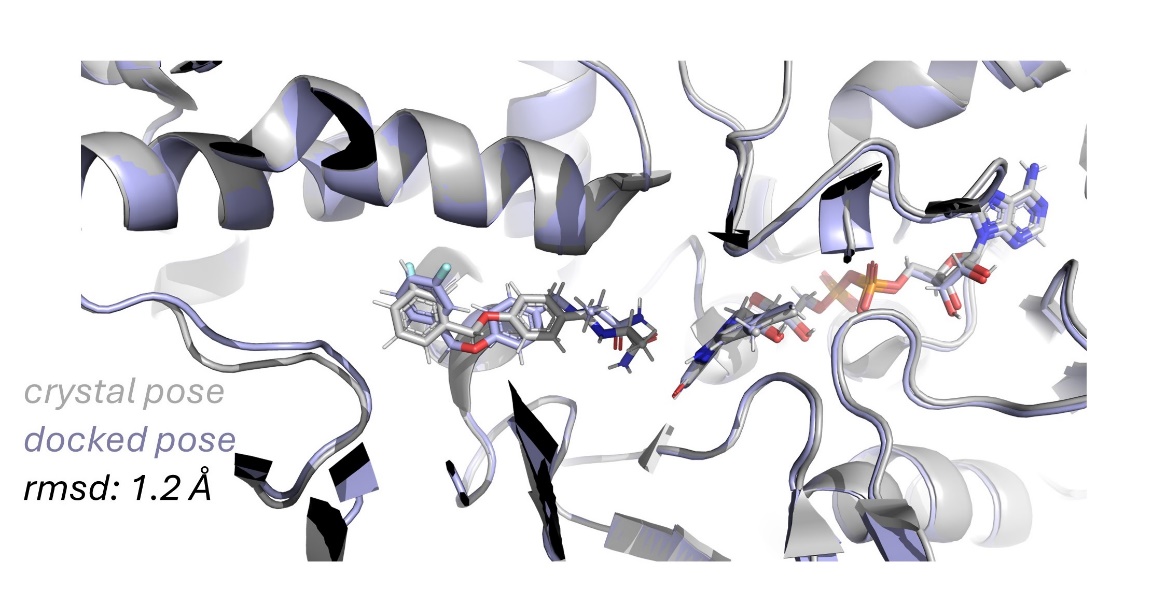
**

***Figure S1.*** *The validation of the docking protocol by superposing the crystal and docked pose of the safinamide.*

**
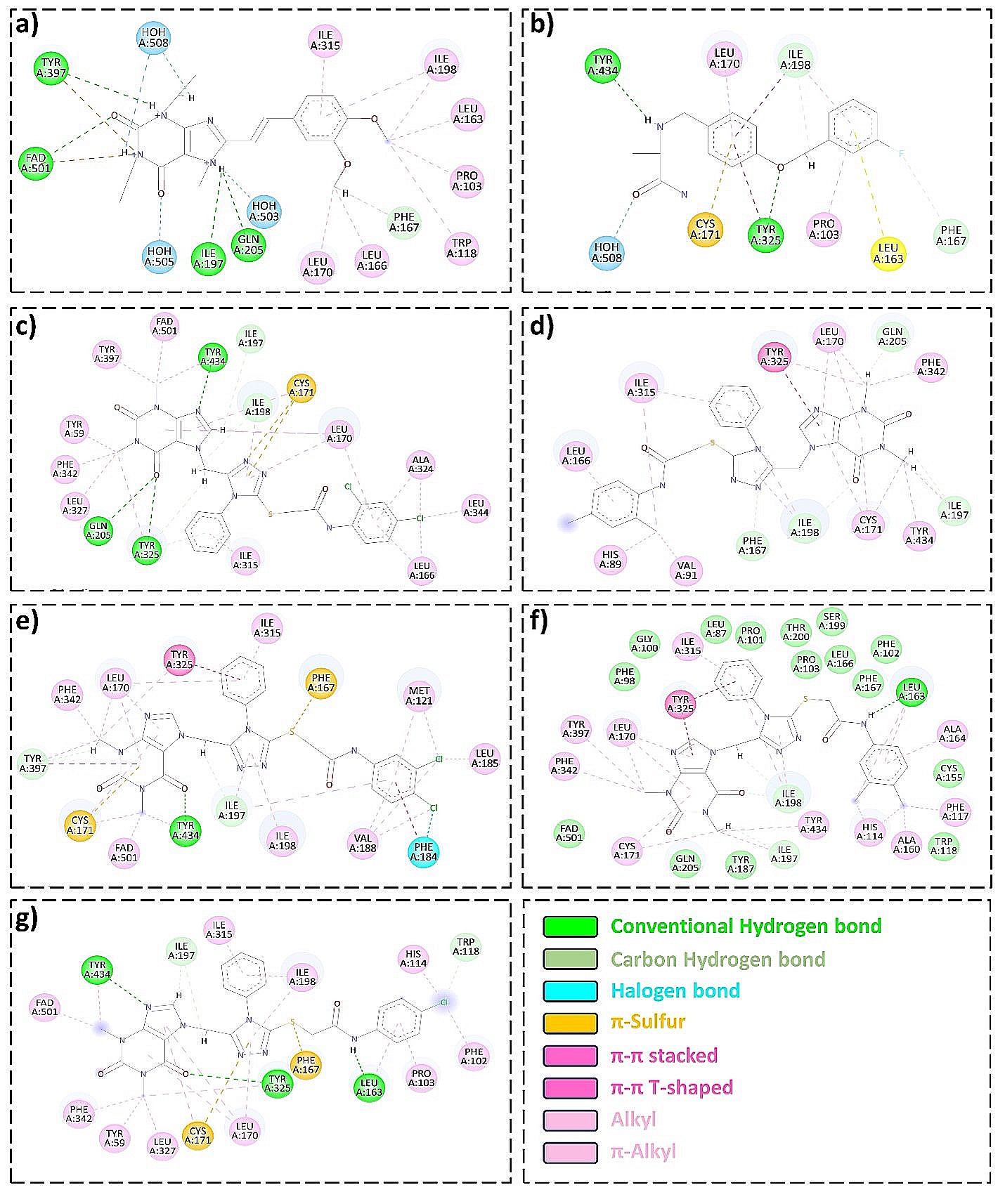
**

**Figure S2.** *The 2D schematic depiction of the molecular interactions of* ***(a)*** *Istradefylline and* ***(b)****,* ***(c)*** *MAO-B14,* ***(d)*** *MAO-B15,* ***(e)*** *MAO-B16,* ***(f)*** *MAO-B20, and* ***(g)*** *MAO-B21 with detailed information regarding the type of hydrogen bonding and hydrophobic interactions. The hydrogen interaction is shown either as the conventional hydrogen bonds or the carbon hydrogen bonds while the Hydrophobic interactions are shown as alkyl, π-alkyl, π- π stacked etc. the color key explains the different type of interaction shown in the figure.*
